# Supplementary figures and images for: A qualitative study exploring the process of postmortem brain tissue donation after suicide
Source: Sci Rep. 2022 Mar 18;12:4710. doi: 10.1038/s41598-022-08729-5 (PMC8933424; doi:10.1038/s41598-022-08729-5)

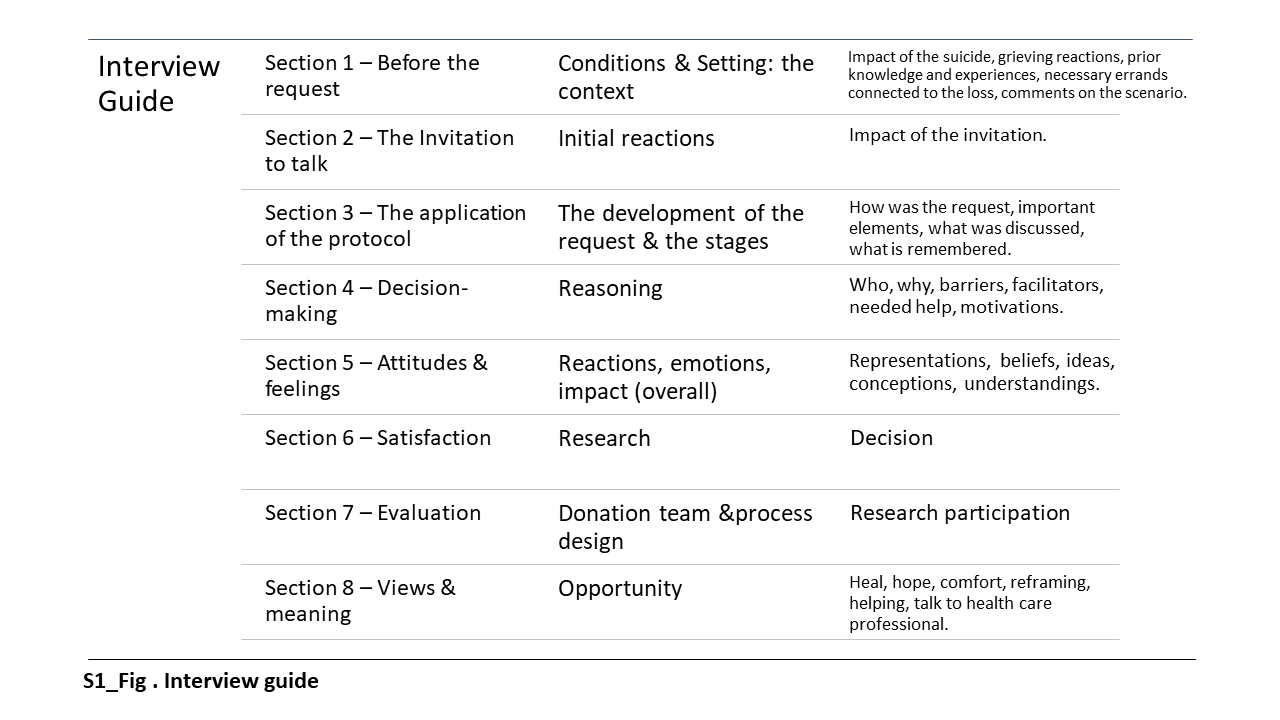

Supplement: Supplementary file 1 — Supplementary Information 1. [file 41598_2022_8729_MOESM1_ESM.tif]
